# Learning from COVID-19? An environmental mobilities and flows perspective on dynamic vulnerabilities in coastal tourism settings

**DOI:** 10.1007/s40152-021-00242-1

**Published:** 2021-09-14

**Authors:** Machiel Lamers, Jillian Student

**Affiliations:** Environmental Policy Group, Wageningen University, Wageningen, the Netherlands

## Introduction

The global COVID-19 pandemic has been claimed to be the most dramatic shock the international tourism and travel sector has faced in the post WWII period (Hall et al. 2020; UNWTO 2020), and the implications are heavily debated since. COVID-19 has severely reduced and in many places stopped tourism mobility (e.g. Gössling et al. 2020; Gretzel et al. 2020; Sigala 2020). Societal and academic debate focuses on how the tourism industry can respond to and recover from this crisis and, ultimately, how travel and tourism will evolve as a socioeconomic activity in our society (e.g. Guardian 2020; Hall et al. 2020; Higgins-Desbiolles 2020; Jamal and Budke 2020).

In addition to more practical recovery or post-crisis questions and debates, COVID-19 has highlighted the inherent vulnerability of the tourism and travel sector and the communities dependent on transnational tourist flows (e.g. Assaf et al. 2021). It has become clear that global transportation and travel flows have played, and continue to play, a central role in the spread of the virus, at a rate and scale that seems unprecedented in history. Tourism strongly contributed to, and is heavily affected by, the pandemic. This inspires us to explore the key role of dynamic transnational tourism flows in generating dependency and vulnerability.

COVID-19 has also affected marine and coastal destinations. The predictable flows of airborne and cruise tourists dissolved completely, with great uncertainties about their foreseeable return (Gössling et al. 2020; Gretzel et al. 2020; Sigala 2020). In general terms, since the start of the pandemic, we have observed difficult policy decisions in many coastal tourism destinations, balancing between lockdowns and border closings for the sake of public health, and attempts to partly or temporarily open up again for the sake of the economy and local livelihoods. Worldwide, marine and coastal destinations are also particularly dependent and thereby vulnerable to global environmental change and dynamic tourist flows (Becken 2013; Leposa 2020; Student et al 2020).

Vulnerability of and from tourism crises are increasingly discussed in the academic literature, in the context of terrorism, war, social unrest, financial and economic crisis (e.g. Blake and Sinclair 2003; Hall 2010; Jóhannesson and Huijbens 2010; Leposa 2020; Sönmez et al. 1999), as well as in the context of the impacts of global environmental change on tourism destinations (e.g. Calgaro et al. 2014; Student et al. 2020). In relation to climate change, the definition of the term vulnerability has been subject to debate (Adger 2006; Eakin and Luers 2006; Gallopín 2006; IPCC Working Groups I & II 2012; Scott et al. 2019; Schröter et al. 2005). A commonly used definition is the one of the 2007 IPCC report, which states that ‘[v]ulnerability is the degree to which a system is susceptible to, and unable to cope with, adverse effects of climate change, including climate variability and extremes. Vulnerability is a function of the character, magnitude, and rate of climate change and variation to which a system is exposed, its sensitivity, and its adaptive capacity’ (IPCC 2007: 21).

Coastal regions tend to be places where many of these global environmental changes and shocks meet, including sea level rise, biodiversity loss, drought and extreme weather events, but they are also places that tend to be highly dependent on tourism, thereby creating many uncertainties and vulnerabilities (e.g. Becken 2013; Student et al 2020). However, COVID-19 demonstrates that the list of global shocks and stressors impacting on coastal and marine tourism destinations is broader than the well-known rapid onset phenomena associated with climate change, political conflict or economic crises. The dynamic and oftentimes combined nature of these crises suggests that assumptions of static ‘stocks’ of exposure, sensitivity and adaptive capacity are insufficient for understanding vulnerability and developing adaptation strategies that address the changing situation. Valls and Sarda (2009) claim that tourism destinations will have to manage constant and increasing uncertainty. In other words, destination vulnerability has a dynamic nature (Adger 2006; Smit and Wandel 2006; Turner et al. 2003; Student et al. 2016). Vulnerabilities typically emerge as a result of multiple conditions, impacts and (non)responses manifested over time (Student et al. 2020). Phillips et al. (2020) state that ‘as outbreaks continue, governments will be faced with developing and adjusting policies that address not only the pandemic itself, but also potential collisions and intersections with other regional or global crises’ (Phillips et al. 2020: 586). It is therefore critical for researchers and decision-makers to consider changes to exposure, sensitivity and adaptive capacity.

Much has already been written about short-term effects of COVID-19 on tourism and projections about long-term implications (e.g. Gössling et al. 2020; Hall et al. 2020; Falk and Hagsten 2020; Sigala 2020). However, the situation around the COVID-19 pandemic’s emergence and persistence has the potential to highlight some of the existing tourism-related environmental challenges in the marine and coastal environment beyond the pandemic. For example, Gretzel et al. (2020) use the pandemic to reflect on transformative research for eTourism, while Higgins-Desbiolles (2020) argues that the pandemic is an opportunity to rethink neoliberalism in tourism practices. COVID-19 also provides a unique opportunity to assess the positive and negative environmental implications of destinations without flows of tourists (e.g. Rutz et al. 2020), and it brings attention to how the flows of people (e.g. tourists) and the environment combine in ways that induce locally manifested vulnerabilities. The spread of COVID-19 helps identify the integrated and dynamic nature of global interactions, change and impacts in tourism. It has made clear that regarding the pandemic as a local or national problem will not provide the necessary insights to identify effective solutions. The focus on single societal or geographical units is insufficient to prevent or respond to these types of challenges; understanding the interconnecting flows is essential to develop adaptive policies. We argue that in order to better understand this uncertain and emergent nature of vulnerability, new theoretical perspectives describing interrelations between local marine environments and global tourist flows are needed. Therefore, we will draw on the social theory literature on environmental mobilities and flows to address these global–local interconnections.

In this paper, we aim to build a conceptual framework for understanding dynamic environmental vulnerabilities in marine and coastal tourism destinations. We are particularly interested in the dynamics and interrelations between various tourism-related flows in the emergence of environmental vulnerability. This conceptual paper is primarily based on existing literature on the sociology of environmental mobilities and flows and tourism vulnerability, previous research on the vulnerability of tourism in the Caribbean region, as well as on recent academic commentaries and examples on the impact of COVID-19 on tourism in coastal settings. It seeks to explore and extend the potential of the environmental mobilities and flows perspective to understand the dynamic nature of vulnerability of tourism in coastal and marine settings.

The paper is organised as follows: We first introduce the main features of the environmental mobilities and flows perspective and discuss previous research on coastal and marine tourism in which this perspective has been applied. Then, we introduce four types of tourism and environmental flows that the global pandemic has revealed. This section describes the main characteristics of the flows, provides an illustration of these four flows in the coastal and marine setting and highlights implications for vulnerabilities. We conclude this paper with a discussion on governance implications of the environmental mobilities and flows perspective in understanding dynamic marine vulnerabilities, and we indicate potential avenues for future research.

## Environmental mobilities and flows

Sociological understandings of environmental mobilities and flows aim to address the social and environmental implications of globalisation (Boas et al. 2018; Mol and Spaargaren 2012; Oosterveer 2018). Based on social theorists like Beck, Castells and Urry, mobilities and flows aim to push away from nation-states or communities as central units of analysis, to a greater attention for the flows of finance, people, information, images and materials and the transnational networks of actors, organisations, institutions and places that enable and constrain the movement of these flows. Environmental mobilities refer to the movements of human actors and non-human entities and the environmental factors and impacts associated with these movements (Boas et al. 2018). The environmental mobilities and flows perspective thereby aim to focus analyses on interconnections and dynamic interactions between local and global phenomena and their environmental implications.

The environmental mobilities perspective builds on the ‘mobilities paradigm’ (Sheller and Urry 2006) in the social sciences or similar traditions like the sociology of networks and flows (Spaargaren et al. 2006). A great inspiration has been the notion of flows as part of Manuel Castells’ work on the network society (Castells 1996), which due to its initial application on the Internet defined the term flows as ‘streams of information between nodes, circulating through the channels of connection between nodes’ (Castells 2009: 20). Later on, sociologists expanded these flows to include streams of people, goods, finances, knowledge, information and energy (Spaargaren et al. 2006; Urry 2016). In that light, mobilities or flows can be conceptualised as material and non-material entities, moving and connecting across time and space, between different nodes in a network. The inclusion of material flows has increased their relevance for analysing contemporary environmental problems, including studies on carbon-based mobility systems such as aeromobility, automobility and shipping (Urry 2016), on the sustainability of (urban) mobility systems and infrastructures (see Freudendal-Pedersen 2009; Jensen and Lanng 2016), on the global financial flows for nature conservation (Anyango-Van Zwieten et al. 2019) and on the governance of various material flows, including biofuel (Oosterveer 2015).

The environmental mobilities and flows perspective bring a range of important and relevant features to help explain contemporary social and environmental phenomena (Mol 2010; Oosterveer 2018). First, this perspective offers ‘a new kind of time–space organisation of practices is introduced that takes globalisation fully into account’ (Mol 2010: 29). Globalisation should not be understood as a spatial dimension separate or in opposition to the local but as an increasing interconnectivity between different localities. Second, the material and the social should not be understood in isolation but integrated with hybrid concepts that acknowledge the social dimension in materiality and vice versa. Material environmental flows should be understood in terms of their interactions with social structures, institutional arrangements and governance structures (Oosterveer 2018). Third, there is an increasing understanding that environmental mobilities and flows can be notoriously challenging to govern from a nation-state perspective and requires diverse networks of actors, organisations and institutions, involving a mix of state, market or civil society interests (Boas et al. 2018).

In order to understand vulnerabilities from environmental mobilities and flows, one needs to start with examining *what* is moving and *how* it moves. For example, for analytical purposes, Urry (2003) makes a distinction between predictable and stable flows moving through integrated networks and unpredictable fluids. Other authors have extended the range of analytical characteristics of mobilities and flows in terms of their motive force, speed, rhythms, routes and frictions (Cresswell 2010) or by analysing the material, social, spatial and temporal dimensions of environmental mobilities and flows (Boas et al. 2018). These characteristics are crucial for understanding vulnerabilities associated with these environmental mobilities and flows. For example, environmental mobilities may create uncertainties and be hard to observe and control due to the fast or slow speeds at which they move or due to the combined character of their effect.

## Tourism and environmental mobilities

The conceptual lens of mobilities holds potential for understanding tourism mobilities and its sustainability challenges. For example, it has been argued how regular tourism mobilities create dependencies in destinations (Williams 2013) and how mobilities of tourists, residents, capital and imagery should be explicitly considered in sustainable destination planning (Dredge and Jamal 2013). In the context of remote nature-based tourism destinations, Ruiz et al. (2019) have argued that flows of tourists to and in protected areas are steered from diverse networks of state and non-state actors and how this challenges spatially bounded forms of governance. Similar conclusions were drawn in the context of cruise ship mobilities to, and cruise tourist mobilities on, Caribbean islands. Van Bets et al. (2017) found that the protocols for negotiating ports of call of cruise ships and activities undertaken by cruise tourists while moored at Caribbean islands are largely determined by the regional cruise industry association, while island actors have limited power (Van Bets et al. 2017).

Studies inspired by environmental mobilities and flows have focused mostly on ways to steer or control predictable flows of tourists with an eye on realising sustainable development goals (e.g. Oosterveer 2018; Mol and Spaargaren 2012). The uncertain, unpredictable, invisible and uncontrollable character of environmental mobilities and flows and their ability to affect the vulnerability and resilience of marine and coastal tourism destinations, which COVID-19 has made us see more clearly, have not received as much attention. We argue that this is due to two reasons. The first reason is that flows of transnational tourists to marine and coastal destinations have been largely taken for granted and considered to be stable and predictable. The second reason is that transnational tourism flows seldomly move alone; they are often interconnected with other flows, compounding or supporting each other, using the same infrastructure, and impacting localities together. For example, flows of tourists to and within tourist destinations are oftentimes accompanied by flows of people (crew, staff), goods (baggage, aircraft, vehicles), food (salmon, beef, bread), water, energy (fuel, electricity) and information (apps). These flows are interconnected in more or less stringent ways, such as through regional agreements between the agricultural and hospitality sectors on the use of fresh water along the Spanish coast (Ricart et al. 2019), through water footprints associated with tourism in the Eastern Mediterranean Sea (Hadjikakou et al. 2013) or through food miles integrated in food products supplied through global supply chains to ecotourism destinations in Fiji (Pratt 2013). These examples make clear that such interconnections between tourism and environmental flows hold important implications for the sustainability profile of tourism activities and developments and for understanding the vulnerability of tourism and its environmental resource base in coastal destinations.

## Tourism flows and environmental vulnerabilities: a typology

**Fig. 1:**
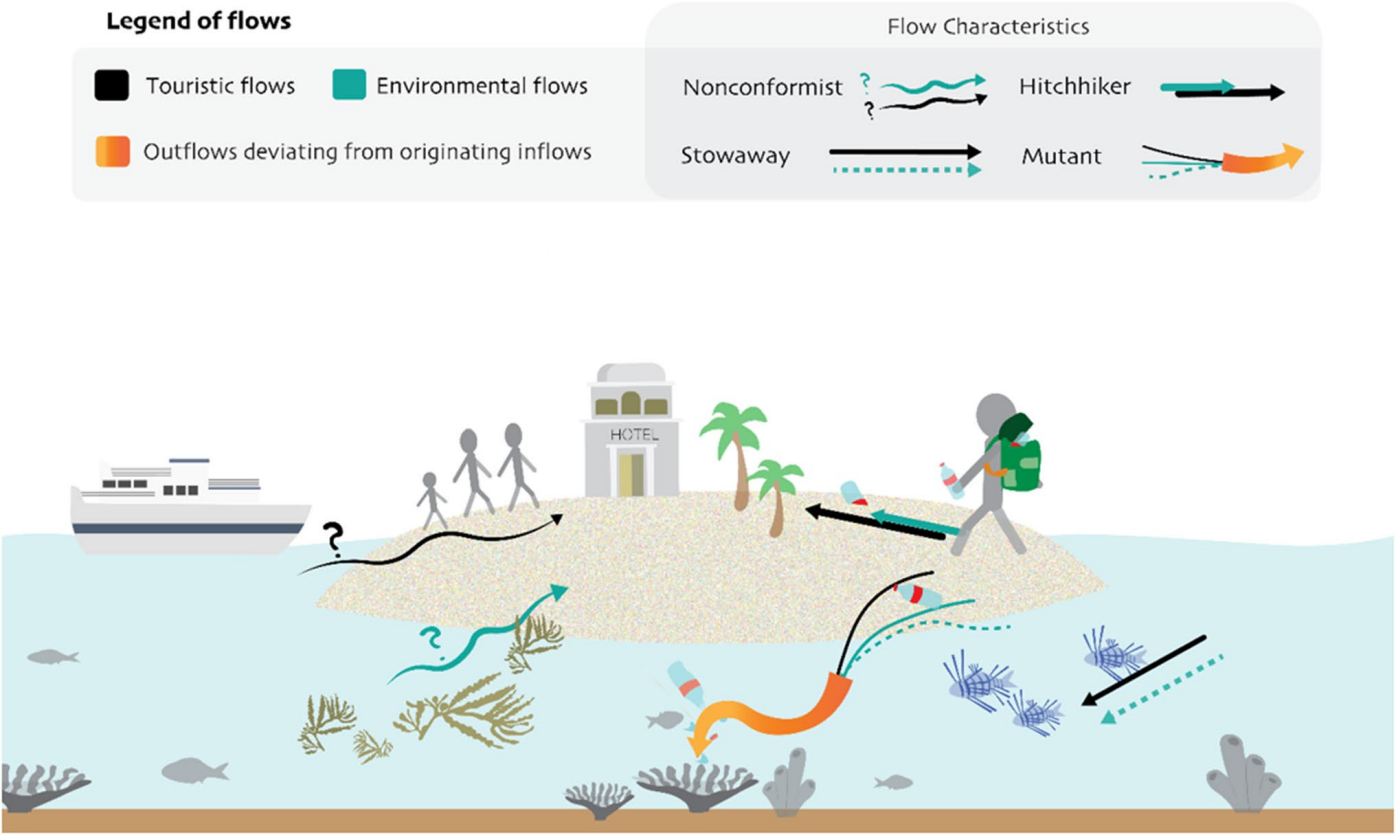
Interconnected environmental and tourism flows in the coastal marine setting

Building on the environmental mobilities and flows perspective, we argue that the dynamic character of the environmental flows associated with marine and coastal tourism and their interrelations result in a variety of modes, whereby for each mode different vulnerabilities emerge. We identify four types of interrelated flows critical to the vulnerability of tourism in coastal and marine environments: nonconformist, hitchhiker, stowaway and mutant. In the subsequent sections, a brief definition and description of each of these interconnected flows are presented, followed by an illustration related to tourism in marine and coastal settings and the related vulnerabilities. Figure 1 provides a visual impression of how these flows can manifest themselves in a coastal destination. In addition, Table 1 depicts these four flows, their visibility, the level of control, their uncertainty, their consequences for the marine environment and their implications for vulnerability. The following examples and illustrations are provided with the aim to clarify our typology, not as an empirical basis to draw conclusions.

**Table 1:**
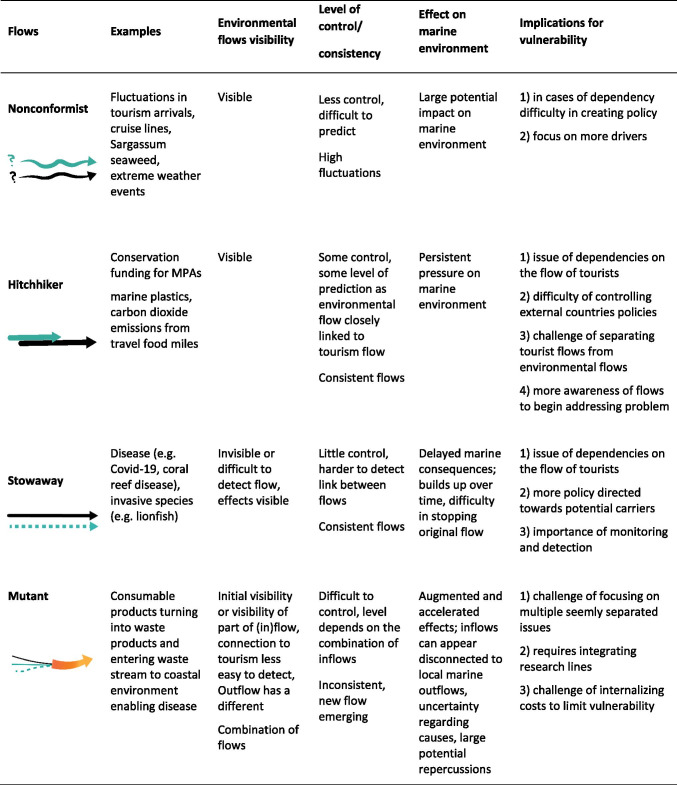
Tourism-environmental flows revealed through the COVID-19 pandemic

### Nonconformist

#### Description

Nonconformist flows are visible flows characterised by uncertainty and high fluctuation. Uncertainty can be in relation to the timing and duration of the flow, while fluctuation refers to dynamic changes to the volume of the flow. Moreover, there is less control over the source or drivers of the flow.

There is a strong relation between international tourist flows and the spread of the COVID-19 virus when viewed from a mobilities and flows perspective. Gössling et al. (2020) noted that UNWTO estimated that global tourist flows in 2020 would decrease to 20–30% of what they were in 2019, which goes against the general global trend of year-on-year increase. In the months since the Gössling et al. (2020) article was published, the uncertainty of tourism flows has only increased: borders have been closed with sometimes very little notice (e.g. the USA), countries and regions have been removed, added and subsequently removed from safe lists (e.g. travel corridors for the UK) and planned travel bubbles have been delayed (e.g. Australia and New Zealand). In May, UNWTO (2020) stated that after a 21% decrease in international tourism during the first quartile, the projection for 2020 is a 60–80% decline depending on when international borders would gradually reopen. Since then, there is persistent uncertainty of when, for whom, how and under what conditions borders will reopen. COVID-19 has revealed the dependence and limited control of destinations on stable tourism flows. Moreover, this reduction and uncertainty in global mobility have direct impact on the regional and local businesses dependent on tourism, including on islands in the Caribbean (e.g. BBC 2020; CNN 2020).

#### Marine illustration

Next to coastal tourists, cruise ships are an example of a nonconformist tourism flow. Higgins-Desbiolles (2020) identifies some of the implications of cruising on the marine environment that have been highlighted during COVID-19: contributing to overtourism and thus pressure on visited destinations and using flags of convenience to avoid environmental regulation. At the same time, the footloose and non-committed character of cruising activities may also entail that cruise ships may suddenly decide to stop frequenting a particular destination.

Several authors outline the general waste streams cruise ships generate—black water, grey water, solid waste, hazardous chemicals, ballast water and air pollution both on-board and during intensive visits to particular destinations. The resulting emissions and waste streams impact coastal destinations and marine areas (e.g. Brida and Aguirre 2008; Lamers et al. 2015).These effects are particularly difficult to regulate by states (Boas et al 2018; Lamers et al. 2015; Van Bets et al. 2017).

An example of an ecologically driven marine nonconformist flow related to coastal tourism is *Sargassum* seaweed—a brown floating seaweed—that since 2011 started inundating beaches and covering nearshore waters in the Caribbean, Brazil and Africa in unprecedented volumes (e.g. van Tussenbroek et al. 2017). While *Sargassum* has affected many Caribbean islands, it has done so irregularly and inconsistently, leaving many uncertainties related to source(s), timing and possible repetition of *Sargassum* flows (Wang et al. 2019). To illustrate, in 2015, large volumes of *Sargassum* were deposited in several Caribbean coastlines (van Tussenbroek et al. 2017; Wang et al. 2019). When tourism operators in Barbados were asked how *Sargassum* affected their operations in the summer of 2015, there were a variety of responses: some mentioned that it had increased costs of operations to remove the seaweed; some had less tourists visiting their areas of the beach and marine area resulting in lost revenue; some were concerned with how and if they would need to prepare for large deposits of *Sargassum* in the future; and other coastal tourism operations were aware of *Sargassum*, but not affected by it directly that summer. This is in contrast to responses from tourism operators in Curaçao in 2016, an island which was not affected by large deposits of *Sargassum* in 2015. Respondents did not express concern about *Sargassum* affecting their beaches and marine areas.

#### Implications for vulnerabilities

A dependency on nonconformist tourism flows (tourists, cruise ships) makes destinations economically vulnerable. At the same time, the uncertainties associated with nonconformist flows pose a challenge for predicting and governing implications of these flows. In the example of *Sargassum* flows, uncertainty may not only increase the economic burden of tourism operators and deter tourism flows. Large deposits of decomposing *Sargassum* also release toxic gases that can form serious human health hazards (Resiere et al. 2018). While *Sargassum* can act as a nursery for marine life, in the short-term, large masses in nearshore waters reduce light, pH and oxygen, leading to mortality of sea grass, nearshore corals and associated marine life and in the long-term can lead to increased turbidity, loss of biomass, eutrophication and increased vulnerability to hurricanes and storms (van Tussenbroek et al. 2017). Wang and Hu (2017) suggest a tracking method to relieve some of the burden of shore removal of *Sargassum* and provide early warning signals to prepare for incoming flows. In addition to this, improving scientific understanding of the drivers of these flows and of the combined effects for coastal livelihood and marine life is necessary to limit vulnerabilities to this emerging environmental challenge.

### Hitchhikers

#### Description

Hitchhikers are visible environmental or socioeconomic flows that accompany tourism flows (e.g. tourists, ships), which makes them relatively easy to monitor and predict. They may be considered a known and observable by-product of tourism. Some hitchhiking flows may be desired, such as monetary flows, while others have negative implications, such as CO_2_ emissions, a known environmental hitchhiker that tags on tourism (e.g. Gössling and Scott 2018). Being aware of desired or undesired hitchhikers provides a window of opportunity to steer or block them. For example, a common initial response to COVID-19 has been one of closing borders, locking down or disconnecting oneself from international travel networks, social distancing and personal hygiene, in order to minimise the virus’ chances to spread, while later on the economic consequences of decoupling were felt. The pressure to reinstate tourism flows for economic recovery exposes both the sending and receiving countries to adverse flows of the virus (e.g. Farzanegan et al. 2020).

#### Marine illustration

Tourists bring with them flows of funding to coastal destinations to support the economy, as well as conservation efforts. Marine protected areas are part of the global strategy to conserve marine biodiversity (e.g. Balmford et al. 2004). However, marine protected areas are costly to set up (McCrea-Strub et al. 2011) and maintain (Balmford et al. 2004). Tourism flows have been a way to fund biodiversity conservation efforts in marine protected areas and provide some alternative sources of livelihood in local coastal communities (e.g. Pham 2020). However, these funding flows have decreased in the absence of tourist arrivals. COVID-19 has jeopardised marine parks’ operating budgets to monitor coral reefs and prevent illegal fishing (UNESCO 2020). In other words, without the tourist flow, the monetary flow for supporting the conservation of marine life is not able to hitchhike.

In the context of the marine environment, other telling examples of a hitchhiker are the flows of plastic waste that accompany tourist flows (e.g. Gössling and Peeters 2015). Plastic waste flows are not only related to tourism flows, but bringing visitors into an area increases the quantities of food products and other materials (e.g. souvenirs), brought into a destination and the waste flows that result from these material flows. Due to hygiene concerns, convenience and the on-the-move nature of tourist practices, food items consumed by tourists are often wrapped in plastic or consumed using single-use plastic cups, plates and cutlery (see also Portman and Brennan 2017).

#### Implications for vulnerability

Hitchhiking materials tend to increase the environmental burden on marine areas, while financial flows can also ameliorate the impacts. A key governance challenge associated with these flows is limiting or decoupling undesirable environmental or social flows from desirable tourism flows. The dependence on tourism flows for (marine) conversation contributes to marine projects’ vulnerability: there is a need to focus on what tourism brings and how disruption of tourism flows disrupts the projects’ financial flows. The former makes it difficult to limit tourist flows to ensure conservation efforts, while the latter brings uncertainty to the longevity.

At the same time, for undesirable hitchhiker flows, such as plastic waste, efforts are needed so that larger tourist numbers do not necessarily equate larger environmental vulnerability. Thailand has, for example, initiated cigarette and littering bans on some of its beaches to curb the hitchhiking of plastic marine waste (e.g. Marks et al. 2020). Moreover, in studying these linked flows, we can better understand the means, timing and locations of these flows, so that targeted actions, instead of general bans, can be used to limit hitchhikers.

### Stowaways

#### Description

Similar to hitchhiker flows, stowaways accompany tourism flows. However, stowaway flows tend to be completely or partly hidden, invisible or unknown. The environmental or socioeconomic consequences are visible and tend to be delayed and largely unintended. Because of delays between the flow and its consequence, the connection to tourism and transport networks tends to be harder to pinpoint. COVID-19 has increased our awareness of viruses or other undesired items that may be travelling unintendedly with other mobilities or flows. The incubation period of COVID-19 and asymptomatic cases hides the stowaway virus from its source. Scott et al.’s (2016) review of the 5th IPCC report determined that there was not much reflection on the relation of tourism and the spread of diseases (see Apostolopoulos and Sönmez 2007 for an exception). Due to COVID-19, there is a growing awareness of the link between tourism, transport and disease (e.g. Gössling et al. 2020). For example, it was determined that European tourists brought back COVID-19 from their ski vacations in the Alpine countries (Falk and Hagsten 2020). Farzanegan et al.’s. (2020) multiple regression analyses indicated ‘that countries exposed to high flows of international tourism are more prone to cases and deaths caused by the COVID-19 outbreak’ (Farzanegan et al.’s 2020: 1).

#### Marine illustration

Tourism mobilities carry or enable not only diseases in terrestrial but also in marine ecosystems. Increased flows of tourists can cause stress on coral reefs. Van de Water et al. (2015) suggest that although they could not pinpoint the specific anthropogenic flows leading to increased coral susceptibility to disease and damage, the unhealthy corals’ proximity to dive sites warranted further study on anthropogenic sources. Lamb et al.’s (2014) study on the impact of scuba diving in reef sites found that the intensity of scuba diving does correlate with coral disease prevalence, which occurred three times more often at frequently visited dive sites compared with lesser used dive sites. While the study focused on the Asia–Pacific region, similar tourism anthropogenic flows likely contribute to disease elsewhere. The Caribbean, a popular coastal tourism region, has long been considered ‘a “disease hot spot” due to the fast emergence and high virulence of coral reef diseases/syndromes’ (Weil et al. 2006: 1).

In addition to spread of disease, trade and transport are important drivers contributing to a ‘high risk of increased potential future impacts of biological invasions’ (Essl et al. 2020: 4890; see also Hulme 2009). Essl et al.’s (2020) findings suggest that recreation and tourism are important unintentional drivers of invasive species and threats to biodiversity, especially in (sub)tropical regions and emerging economies. In other words, the flow of people and goods contribute to stowaway invasive species.

#### Implications for vulnerability

Due to their hidden character and the delayed visibility of the consequences, stowaways are prolific contributors to coastal and marine vulnerability. Their presence can have long-term weakening effects on the marine environment’s sociocultural, economic or ecological systems. A key governance challenge is therefore to invest in research to be able to detect the presence of stowaways, in order to block their access or to turn them into hitchhikers so that they can be monitored. This necessitates a more holistic approach to looking at the flows of tourists and changes to the environment by examining their correlations, which may be indicative of a stronger relationship than initially assumed.

For example, the reopening of air travel and the tourism market after the initial lockdowns over the Summer of 2020 was strongly connected to increasing capacities to test travellers for COVID-19 on departure and arrival in their destination, taking away some of COVID-19’s ability to be a stowaway flow. Similarly, the ability to monitor changes to marine areas frequented by tourists and characterising these flows in conjunction with changes to indicators of marine health can help to unveil these stowaway environmental flows and convert them to hitchhiker flows.

### Mutants

#### Description

Mutant flows constitute the meeting of two or more flows that contribute or transform to a new and unexpected phenomenon or flow. The inflows can be any combination of the above-mentioned flows, i.e. individual flows may be visible, uncertain, delayed. However, the combination of these flows brings about new consequences that may not be identified by looking at the contributing flows individually. The emergent character of mutants makes them less predictable. COVID-19 emphasises the implications of compounding risks and the need to work on both short-term challenges and long-term planning (e.g. Gretzel et al. 2020; Phillips et al 2020). For example, next to testing and vaccinating the national populations as strategies in the fight against COVID-19, public health authorities are currently concerned about the emerging variants of the virus.

#### Marine illustration

Phillips et al. (2020) show that in addition to responding to COVID-19, some coastal regions and islands are simultaneously exposed to storms, hurricanes or water quantity issues (flooding and drought). A marine-specific example is when the hitchhiker flows of plastic waste meet stowaway flows of coral reef disease. For example, in the Asia–Pacific region, Lamb et al. (2018) found an increased disease susceptibility of reefs in locations where plastics were present. Both flows could very well be associated with tourism. Baker et al. (2013) established the interrelation—or ‘tourism’s nitrogen footprint’—between the volume of tourism flows and waste streams on land and the increased eutrophication levels in the marine environment.

Mutant flows result from, and are dependent on, the nature and combinations of flows that they consist of. For example, eutrophication and pollution flows were found by Essl et al. (2020) as important drivers for alien species in marine contexts. Stony Coral Tissue Loss Disease, a particularly contagious marine disease first detected in Florida in 2014, has been rapidly spreading in the Mexican Caribbean and has ‘increased coral mortality and severely changed the structure of coral communities in the region’ (Alvarez-Filip et al. 2019: 9). The authors suggest that along with pollution flows, increased inundation of *Sargassum* is likely to further decrease water quality, contributing to conditions for the disease to spread and coral coverage to decline. The combination of flows—tourists, plastic waste and eutrophication—leads to new flows, such as the Stony Coral Tissue Loss Disease, which increase the marine environment’s vulnerability.

#### Implications for vulnerability

Mutants, or combinations of flows, may lead to compounding risks, such as in the case of the Stony Coral Tissue Loss Disease. This example further highlights the limitations of focusing on one part of the issue to resolve a marine challenge. Although eutrophication, tourism flows, invasive species and *Sargassum* may seem unrelated, their combined influence threatens the health of Caribbean reefs, which will ultimately threaten socioeconomic activities. An environmental mobilities and flows perspective helps to identify the combinations of human and environmental flows and to the understanding of emerging mutant flows leading to coastal and marine vulnerability.

## Conclusion

In the academic discourse, the short- and longer-term implications of the pandemic have been discussed extensively (e.g. Hall et al. 2020; Cheer 2020). In this paper, we show how COVID-19 can open our eyes to the dynamic nature of vulnerability of tourism in coastal and marine settings, a perspective that has been voiced and operationalised before (Calgaro et al. 2014; Student et al. 2020). In this article, we aimed to build a conceptual framework for analysing and managing dynamic vulnerabilities in coastal and marine tourism resulting from transboundary, fluctuating, uncertain, hidden, delayed or compounding flows. We identified and illustrated four types of interrelated flows that can assist in understanding dynamic vulnerabilities in coastal tourism destinations and beyond, each pointing to different characteristics. The nonconformist flow highlights the unpredictable and fluctuating nature of human mobilities and material environmental flows and the implications of uncertainty and limited control. The hitchhiker points to the visible and intentional interrelation of desirable and undesirable socioeconomic and environmental flows and the challenge of decoupling. Building on the previous type, the stowaway adds an element of invisibility, which is essential for targeted measures and governance approaches. Finally, the mutant points to the tendency of socioeconomic and environmental flows to mix up, combine and form emergent properties, with unpredictable outcomes. These characteristics contribute and correspond closely to what Phillips et al. (2020) have termed compounding vulnerabilities.

The environmental mobilities and flows perspective has been used as an insightful starting point for defining these four types of flows, characterising the mobile and transnational character of societal and environmental issues and identifying the challenges to control and govern such flows effectively (e.g. Oosterveer 2018). We argue that COVID-19 is particularly pointing at the fluidity, uncertainty and unpredictability, as well as the integrated character of different mobile flows, to extend beyond the more common predictable flows in global tourism and travel (Van Bets et al. 2017), food and resource supply chains (Oosterveer 2018) and conservation and climate finance (Anyango-Van Zwieten et al. 2019). Where Urry (2003) is referring to the abundance and diversity of these flows with the single-term global fluids, we argue that a richer terminology is needed to identify and act on these flows. In other words, the pandemic has opened our eyes to the dynamism and invisibility of environmental and socioeconomic flows resulting from tourism mobility, an insight that both tourism scholars and actors managing tourism in coastal and marine settings can further operationalise and act upon after and apart from the current pandemic context.

It should be noted that the current typology is an analytical device, rather than an empirical categorisation, which enables tourism researchers and governance actors to identify and understand a greater variety of unpredictable and volatile environmental mobilities and flows, the associated emerging and dynamic vulnerabilities and the governance strategies to address these vulnerabilities in coastal and marine destinations. We would like to point out that the terminology coined in this article is not an attempt to unnecessarily humanise or grant agency to material environmental flows. With the choice of terminology, we would like to raise awareness of the socio-material character of various combined environmental mobilities and flows (see also Boas et al. 2018; Oosterveer 2018).

The combination of the current COVID-19 crisis with the ongoing climate crisis shows that understanding vulnerabilities as the result of a relatively simple equation of exposure, sensitivity and adaptive capacity misses the multiple interacting factors and the emergent nature of vulnerability. Vulnerability assessments that focus on one source of vulnerability in a static setting do not prepare us enough for the emerging vulnerability challenges, particularly in coastal and marine tourism settings. A dynamic vulnerability approach (Student et al. 2020) shows a means of incorporating different types of local and global environmental challenges in a spatially and temporally dynamic setting.

A key question is how governance actors in coastal and marine tourism destinations can govern the dynamically vulnerable conditions they are facing. First, hitchhiker flows increase marine vulnerability as dependency on single tourist source markets influences marine conservation efforts. In a similar vein, Higgins-Desbiolles (2020) has recently argued that tourism should be reoriented from private interests towards the public good, by providing service and being accountable to the public. Local enterprises should be favoured over multinationals to better serve the destinations and rectify neoliberal injustices.

Second, it is important that we increase our capacities to detect and monitor critical interrelations between flows. Monitoring marine ecosystem changes in combination with tourism flows can expose stowaway flows and make the connection inflows and their effects visible. This essentially converts a stowaway flow into a hitchhiker flow. In addressing stowaways and mutant flows, and their compounding vulnerabilities, these assessments must explicitly consider a diverse spatial and temporal scope, a variety of biophysical, health-related or socioeconomic factors, interdependencies between different sectors (e.g. the food–energy–water–health nexus) and the potential for feedback loops (Phillips et al. 2020). This may require new conceptual tools, signal indicators and metrics, as well as the development of monitoring platforms or dashboards for all kinds of decision-makers, from formal authorities to non-governmental organisations and tourists. Monitoring systems can and should thereby stimulate social learning among stakeholder groups to become more aware of each other’s role in the emergence of, and in dealing with, vulnerability (Student et al. 2020).

Third, based on monitoring results, targeted policy measures could be taken to decouple desired from undesired hitchhiker and stowaway flows and to control and reduce their impact. The ability of a socioecological system to deal with external shocks, like fluctuations in visitation, a pandemic or extreme weather events, may be cushioned by the ability of governance actors in a destination to manage impacts locally (see also Scheffer et al. 2015).

Fourth, there is a need for coordination between different levels of government, as well as public–private partnerships and networks, in governing tourism, to prevent potential conflicts of strategy across agencies, sectors and scales in regulating interconnected flows. This way, governance arrangements can become more integrated and adaptive, considerate of interactions and trade-offs.

Based on our discussion of COVID-19 in coastal and marine destinations, we call for more research aimed at developing and applying conceptual and hands-on methodological tools for analysing how critical flows underlying vulnerability and resilience can be detected, measured, monitored and governed in an integrated way. COVID-19 demonstrates that tourism flows should not be ignored when looking at global issues. We argue that it is critical to link tourism flows to environmental flows in order to limit vulnerabilities to the marine environment. The sociology of environmental mobilities and flows provides a promising avenue in this regard.

## Data Availability

Not applicable.
